# The Efficiency of Respiratory-gated ^18^F-FDG PET/CT in Lung Adenocarcinoma: Amplitude-gating Versus Phase-gating Methods

**DOI:** 10.22038/aojnmb.2016.7747

**Published:** 2017

**Authors:** Yoshiyuki Kitamura, Shingo Baba, Takuro Isoda, Yasuhiro Maruoka, Satoshi Kawanami, Kazuhiko Himuro, Masayuki Sasaki, Hiroshi Honda

**Affiliations:** 1 Department of Clinical Radiology, Graduate School of Medical Sciences, Kyushu University, Fukuoka, Japan; 2 Department of Molecular Imaging and Diagnosis, Graduate School of Medical Sciences, Kyushu University, Fukuoka, Japan; 3 Division of Radiology, Department of Medical Technology, Kyushu University Hospital, Fukuoka, Japan; 4 Department of Health Sciences, Graduate School of Medical Sciences, Kyushu University, Fukuoka, Japan

**Keywords:** FDG-PET/CT, Lung adenocarcinoma, Positron emission tomography, Respiratory gating

## Abstract

**Objective(s):**

In positron emission tomography (PET) studies, thoracic movement under free-breathing conditions is a cause of image degradation. Respiratory gating (RG) is commonly used to solve this problem. Two different methods, i.e., phase-gating (PG) and amplitude-gating (AG) PET, are available for respiratory gating. It is important to know the strengths and weaknesses of both methods when selecting an RG method for a given patient. We conducted this study to clarify whether AG or PG is preferable for measuring fluorodeoxyglucose (FDG) accumulation in lung adenocarcinoma and to investigate patient conditions which are most suitable for AG and PG methods.

**Methods:**

A total of 31 patients (11 males, 20 females; average age: 70.1±11.6 yrs) with 44 lung lesions, diagnosed as lung adenocarcinoma between April 2012 and March 2013, were investigated. Whole-body FDG-PET/CT scan was performed with both PG and AG methods in all patients. The maximum standardized uptake value (SUV_max_) of PG, AG, and the control data of these two methods were measured, and the increase ratio (IR), calculated as IR(%)= (Post – Pre)/Pre × 100, was calculated. The diameter and position of lung lesions were also analyzed. We defined an ‘effective lesion’ of PG (or AG) as a lesion which showed a higher IR compared to AG (or PG). 8 (25.8%)

**Results:**

The average SUV_max_ and average IR were 8.99±7.94 and %21.4±25.6 in PG and 7.60±6.70 and %4.0±14.4 in AG, respectively. Although there was no significant difference between the average SUV_max_ of PG and AG (P=0.09), the average IR of PG was significantly higher than that of AG (P<0.01). The number of PG- and AG-effective lesions was 32 (72.7%) and 12 (28.3%), respectively. There was no significant difference in the average diameter or position of the lesions between the PG- and AG-effective lesions. There were 23 (74.2%) PG-effective and 8 (25.8%) AG-effective patients. No significant difference was observed in sex or age between PG- and AG-effective patients.

**Conclusion:**

The PG method was more effective for measuring FDG accumulation of lung lesions under free-breathing conditions in comparison with the AG method.

## Introduction

Thoracic movement under free-breathing conditions is a cause of image degradation in positron emission tomography (PET) studies (1). Respiratory movement during PET/computed tomography (CT) examinations may result in the misalignment of fused PET/CT images, leading to attenuation correction errors and mislocalization of fluorodeoxyglucose (FDG) uptake. Breath holding by the patient can solve this problem, although a rather long period of breath-holding is needed during scanning, to obtain a sufficient photon count which is not achievable by all patients.

Respiratory-gating (RG) methods have been recently introduced to solve the discussed problem. Phase-gating (PG) (2) and amplitude-gating (AG) (3) PET studies are major RG methods for clinical use. Both PG and AG methods are feasible and effective in the measurement of FDG uptake by extracting the gated data. This is usually accomplished by simultaneously recording a respiratory wave signal of the patient with the PET data.

The PG method is often used with respiratory tracking systems to monitor the respiratory motion during PET scans. The PET data are sorted into discrete bins, corresponding to certain time intervals in the respiratory cycle. The PET images, reconstructed from each of the bins, correspond to a phase of the cycle. In the AG method, the PET data are sorted into bins according to the respiratory amplitude range. This range is determined by analyzing the respiratory amplitude histogram.

Kawano et al. suggested that breath-holding could effectively result in an improvement in image degradation (4) however, not all patients can do breath-holding easily or for a long period of time for the acquision of PET data. Both PG and AG methods can be applied for almost all patients, and the control of breathing training is not required.

Several studies have demonstrated the advantages of RG in free-breathing acquisition using the PG (5-8) or AG (3, 9) method. In our institution, PG requires a longer scan period than AG, as AG can be integrated in the whole-body (WB) scan seamlessly, whereas PG cannot. For many institutions which incorporate RG in their routine scans, it is important to know the differences in the effects of PG and AG when selecting an RG method for a given patient. However, few studies have directly compared the effects of PG and AG methods on the measurement of FDG accumulation (10, 11); also, the possible differences in these effects have not been identified.

In this study, we applied both methods for the same patients in a single FDG-PET/CT study. Our two main objectives were to establish which method is more effective in the measurement of FDG accumulation in lung adenocarcinoma and to clarify which patient conditions are suitable for PG or AG.

## Methods

### Patients

A total of 31 patients (11 males, 20 females; average age: 70.1±11.6 yrs) with 44 histopathologically confirmed lung adenocarcinomas, examined between April 2012 and March 2013, were evaluated in this study. All patients underwent WB FDG-PET/CT scan with PG and AG methods.

The lung lesions were classified as either an “upper-lung lesion,” i.e., a lesion located at a level higher than the carina, or a “lower-lung lesion,” i.e., a lesion located at a level lower than the carina. There were 16 upper-lung lesions and 28 lower-lung lesions. The diameter of each lesion was manually measured in the axial diagnostic CT image, using the Response Evaluation Criteria in Solid Tumors (RECIST) methods (12). The characteristics of the patients are summarized in Table 1. This study was approved by the Institutional Review Board of our hospital.

**Table 1:** The characteristics of the patients and lung adenocarcinoma lesions

Male/female ratio	Age (yrs)	Lesion diameter (mm)	Position (upper: lower ratio)
11/20	70.1±11.6	23.6±16.0	16:28

### Data acquisition

PET data were acquired for all patients, using a Biograph mCT system (Siemens Medical Solutions, Knoxville, TN, USA). This PET scanner comprises of three rings with a total of 144 lutetium orthosilicate detectors, covering an axial field of view (FOV) of 16.2 cm and a transaxial FOV of 70 cm in diameter; each block is 4×4×20 mm.

The coincidence time window was 4.1 ns, and the time of flight (TOF) time resolution was 555 ps. The spatial resolution values at 1 and 10 cm were 4.4 and 4.9 mm, respectively. The emission data were acquired in the 3D mode and reconstructed with a 256×256 matrix (3.18×3.18×5.00 mm). The 32-slice CT scan parameters were as follows: 120 kV, 100 mAs (Eff. mAs), a 512×512 matrix, slice thickness of 3 mm, and a transaxial FOV of 500 mm.

PET images were reconstructed using 3D ordered-subset maximization (3D-OSEM) with a point spread function (PSF) and TOF algorithm. The reconstruction parameters were as follows: two iterations, 21 subsets, and a post-filter of 5 mm full-width at half-maximum (FWHM) with CT attenuation correction. All patients fasted for at least 4 h before ^18^F-FDG administration. The injected activity of FDG was four times the patient’s body weight and 251.1±48.2 MBq on average.

**Figure 1:**
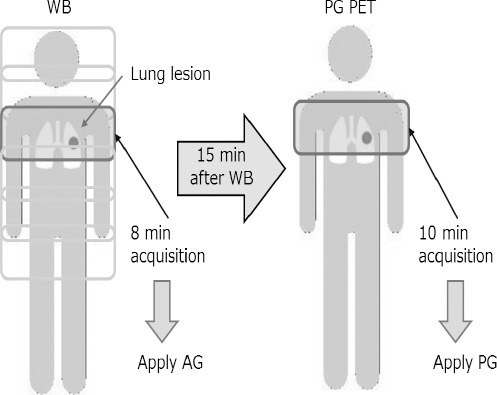
The PET schedule. Scanning was initiated 60 min after an intravenous injection of ^18^F-FDG. First, the whole-body scan which included an amplitude-gating (AG) study was performed. After 15 min of WB scan, a phase-gating (PG) scan was performed in the same bed position as that of the AG study. The acquisition time in the bed position, which included lung lesions, was 8 min for AG and 10 min for PG

The schedule of the PET scan is summarized in Figure 1. Scanning was initiated 60 min after an intravenous injection of ^18^F-FDG. First, the WB scan, including the AG method, was performed accompanied with WB–CT scan for attenuation correction. Second, a PG study was performed in the same bed position as the AG study after 15 min of WB scan, followed by lung CT for attenuation correction. The acquisition time of each bed position including lung lesions was 8 min for the AG-PET study and 10 min for the PG-PET study.

The sequential 2-min acquisition data in the middle time point of PG or AG acquisition time were used as the control data. WB and chest CT scans for attenuation correction were performed between WB and PG scans. The average effective dose from FDG-PET was 4.7±0.9 mSv (13, 14), and the average values of the total dose length product (DLP) in WB and chest CT scans were 295.0±77.3 and 116.4±41.6, respectively.

### Respiratory gating

**Figure 2:**
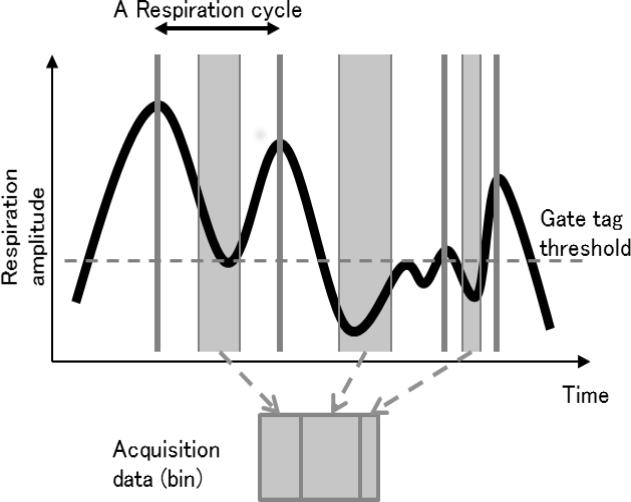
The schematic chart of the phase-gating (PG) method (PG with three gates is shown in this figure for simplification). The local maximum value of the respiration amplitude (gate tag) above the threshold was tagged automatically. One respiration cycle was defined as the time period between two successive gate tags. Each respiration cycle was divided into the determined number of gates and combined for the respiratory-phase data (bin)

The chest wall pressure of the patients was sensed by a respiratory monitoring system (AZ733V, Anzai Medical Co., Tokyo, Japan) during PET data acquisition. The signals of chest wall pressure were converted to a respiratory wave in this system. The PG method is summarized in Figure 2.

Each respiration cycle was divided into five gates as 20% of the cycle phase, and gates of the same number were combined for all respiratory cycles as a phase data (bin). The bin with the highest maximum standardized uptake value (SUV_max_) of the lesions was selected for the PG data. The number of selected bins is summarized in Table 2. The PET data with the same timing in the respiration cycle were collected by this method.

**Table 2:** The number of the selected bins

	Bin 1	Bin 2	Bin 3	Bin 4	Bin 5
Number of lesions (n=44)	2	5	23	9	5

**Figure 3:**
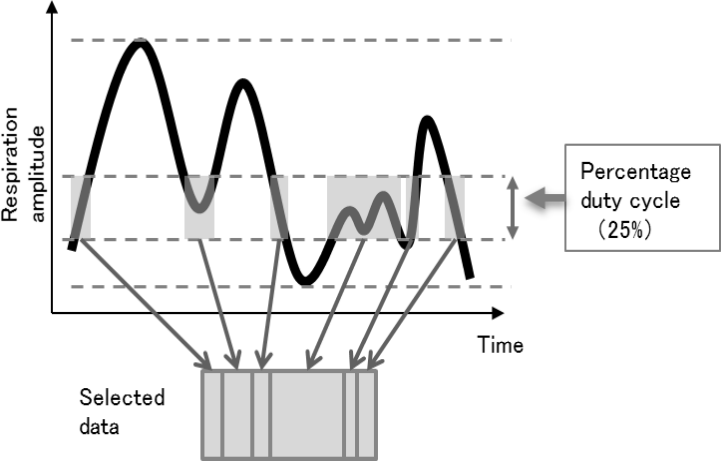
The schematic chart of the amplitude-gating (AG) method. The duty cycle percentage (the percentage of the total acquired true coincidences used for image reconstruction) was primarily determined. The software algorithm calculates an optimal amplitude range for a given duty cycle. The optimal amplitude range is defined as the smallest obtained amplitude range and is calculated by minimizing the range width. In this study, data were reconstructed using duty cycles of 25%

For the non-gated images, the middle time point of 120 s of the acquired data was used for image reconstruction, resulting in an equal amount of the acquired true coincidences as the images reconstructed with the single selected bin. The AG method is summarized in Figure 3. AG was performed, using the list-mode data with an amplitude-based algorithm, integrated in Syngo 2011A MI PET/CT software (HD-Chest) (Siemens Medical Solutions, Knoxville, TN, USA).

In this study, the data were reconstructed using duty cycles of 25%, corresponding to 120 s of the PET data. For the non-gated images, the middle of 120 s of the acquired data was used for image reconstruction, resulting in an equal amount of the acquired true coincidences as the images reconstructed with a duty cycle of 25%.

### Measurements and data analysis

The SUV_max_ was measured by placing a 3D volume of interest on each lung lesion of each PET datum. The increase ratio (IR) was calculated as follows:

IR (%)= (Post-Pre)/Pre×100

where ‘post’ denotes the SUV_max_ of the lesion in PG or AG, and ‘pre’ indicates the SUV_max_ of the lesion in the control data of the PG or AG method.

We defined a PG (or AG) ‘effective lesion’ as a lesion which showed a higher IR compared to AG (or PG). In addition, we defined ‘PG (or AG)-effective patients’ as those who had a PG- or AG-effective lesion, respectively. For patients with multiple lesions, the lesion which had the highest IR of all lesions was used for the calculation of IR in PG and AG to define PG- and AG-effective patients.

Statistical analysis was performed using the statistical software JMP 11 (SAS Institute Inc., Cary, NC, USA). Wilcoxon signed-rank test was used to compare parameters between the control and gated data. Mann-Whitney U test was used to compare lesion diameter and age between PG- and AG-effective lesions. Fisher’s exact test was used to compare lesion position in the lung between PG and AG-effective lesions and sex between PG- and AG-effective patients. P-value less than 0.05 was considered statistically significant.

## Results

The average SUV_max_ values of the control data in PG and AG were 7.92±7.80 and 7.35±6.54, respectively. There was no significant difference in the SUV_max_ of the control data between the PG and AG methods (P=0.09). The average SUV_max_ of the gated data was 8.99±7.94 in PG and 7.60±6.70 in AG. Based on the findings, the average SUV_max_ of PG was significantly higher than that of AG (P<0.01) (Table 3).

**Table 3:** The SUV_max_ and IR values of phase-gating (PG) and amplitude-gating (AG) methods

	PG	AG	P-value
Control SUV_max_	7.92±7.80	7.35±6.w54	0.09
Gated SUV_max_	8.99±7.94	7.60±6.70	<0.01
IR (%)	21.4±25.6	0.6±11.7	<0.01

SUV_max_: Maximum standardized uptake value, IR: Increase ratio [IR(%)= (Post – Pre)/Pre × 100]

**Figure 4:**
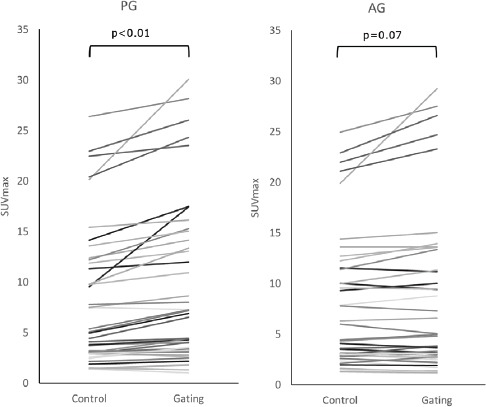
The effects of gating on SUV_max_ in amplitude-gating (AG) and phase-gating (PG) methods. With PG, 37 out of 44 (84.1%) lesions showed increased SUV_max_ after gating. In the same way, 24 out of 44 (54.5%) lesions showed increased SUV_max_ after gating with AG. The increase ratio (RI) tends to be high in lesions with higher SUV_max_ in both methods. There was a statistically significant difference between the SUV_max_ of the control and gated data in PG (P<0.01), while there was no significant difference in AG (P=0.08), based on the paired t-test results

With PG, 37 out of 44 (84.1%) lesions showed increased SUV_max_ after gating. Similarly, 24 out of 44 (54.5%) lesions showed increased SUV_max_ after gating with AG. There was a statistically significant difference between the SUV_max_ of the control and gated data in PG (P<0.01), while there was no significant difference in AG (P=0.07) (Figure 4).

The average IR was 21.4±25.6% in PG and 4.0±14.4% in AG. As it can be seen, the average IR of PG was significantly higher than that of AG (P<0.01) (Table 3). There was no significant difference between the SUV_max_ of the control data of PG and that of AG in the upper (PG, 7.62±7.99; AG, 7.25±7.12; P=0.53) and lower (PG, 8.13±7.83; AG, 7.41±6.33; P=0.10) lesions. The average SUV_max_ of PG was significantly higher than that of AG in both the upper (PG, 9.00±8.74; AG, 7.54±7.59; P<0.01) and lower (PG, 8.99±7.62; AG, 7.64±6.28; P<0.01) lesions.

The average IR of PG was significantly higher than that of AG both in the upper (PG, 23.8±21.9%; AG, 4.1±15.2%; P<0.05) and lower (PG, 20.0±27.8%; AG, 4.4±14.2; P<0.01) lesions (Table 4). There was a statistically significant difference between the SUV_max_ of the control data and that of the gated data in PG both in the upper (P<0.05) and lower (P<0.01) lesions. However, there was no significant difference between the SUV_max_ of the control and gated data in AG both in the upper (P<0.10) and lower (P<0.25) lesions.

**Table 4:** The SUV_max_ and IR values of phase-gating (PG) and amplitude-gating (AG) methods in the upper and lower lesions

	Upper lesions			Lower lesions		

	PG	AG	P-value	PG	AG	P-value
Control SUV_max_	7.62±7.99	7.25±7.12	0.53	8.13±7.83	7.41±6.33	0.10
Gated SUV_max_	9.00±8.74	7.54±7.59	<0.01	8.99±8.62	7.64±6.28	<0.01
IR (%)	23.8±21.9	4.1±15.2	<0.05	20.0±27.8	4.4±14.2	<0.01

SUV_max_: Maximum standardized uptake value, IR: Increase ratio [IR(%)= (Post – Pre)/Pre × 100]

There were 32 (72.7%) PG-effective and 12 (28.3%) AG-effective lesions. The average diameter of the PG- and AG-effective lesions was 24.7±17.4 and 20.3±11.3 mm, respectively. Among 32 PG-effective lesions, the number of upper-lung and lower-lung lesions was 12 and 18, respectively. The corresponding numbers in AG-effective lesions were 3 and 9, respectively. There was no significant difference in the average diameter or position of lesions between the PG- and AG-effective lesions (Table 5).

**Table 5:** The characteristics of phase gating (PG)-effective and amplitude gating (AG)-effective lesions and PG- and AG-effective patients

	PG-effective lesions	AG-effective lesions	P-value
No.	32	12	
Diameter (mm)	24.7±17.4	20.4±11.3	0.74
Position (upper:lower ratio)	12:18	3:9	0.49

	PG-effective patients	AG-effective patients	P-value

No.	23	8	
Age (yrs)	70.9±12.3	67.8±9.8	0.34
Sex (male:female ratio)	7:16	4:4	0.41

There were 23 (74.2%) PG-effective and 8 (25.8%) AG-effective patients. Among the PG-effective patients, there were 7 males and 16 females, while among AG-effective patients, there were 4 males and 4 females. The average age of PG- and AG-effective patients was 70.9±12.3 and 67.9±9.8 yrs, respectively. There was no significant difference in sex or age between PG- and AG-effective patients (Table 5).

## Discussion

In this study, we aimed to investigate the IR of the SUV_max_ of lesions to compare the efficacy of PG and AG methods because it is independent at the volume of FDG avid lesion which is likely to change by using RG methods. In our study of lung adenocarcinoma lesions, the average IR of PG was higher than that of AG, which suggests that PG was more effective than AG in measuring FDG accumulation.

In addition, we evaluated the same findings in the upper and lower lesions. The analysis suggested that RG methods, especially PG, for lung adenocarcinoma lesions under free-breathing conditions should be applied independently of the position of lung lesion.

Two studies have directly compared PG and AG in clinical use (10, 11). Elmpt et al. showed no significant difference between PG and AG in measuring FDG accumulation when applied to the same acquisition data (10); in their investigation, the total length of data acquisition was 24 min. They determined the PG data as the single phase data out of eight phases and the AG data were considered as 35% of the same acquisition data. Therefore, the acquisition time of PG and AG was equivalent to that of 3-min and 8-min of the original data, respectively.

In the mentioned study, there was a significant difference between the two methods. Jani et al. found that AG was more effective than PG in determining the internal target volume of lung tumors (11); however, the FDG avidity of PG and AG was not mentioned in their report.

We noted that the acquisition time of PET data should be the same to compare the tracer uptake between AG and PG methods. We divided 10 min of the acquisition data into five phases to obtain the PG data, while the AG data were determined as 25% of the total 8-min acquisition data. The total acquisition time in the two methods was consequently equivalent to that of a 2-min free-breathing scan. This is one of the reasons why the IR of the PG method was higher than that of AG in our study.

Another reason is the difference in data collection algorithms. AG data contain data of multiple respiratory phases which may result in a more dispersed localization of tumor. On the other hand, RG data were obtained in a more restricted phase of the respiratory cycle. In general, AG assumes that the respiratory amplitude and the corresponding position of the lung lesion are constant in the respiratory cycle. However, they are speculated to change by cycle and might result in a more disperse location of lung lesion in AG. Overall, different amplitude ranges by the respiratory cycle may be needed for optimal gating in AG.

Approximately 70% of the patients were PG-effective in this study, suggesting that PG should be used as the primary RG method for lung adenocarcinoma patients. However, there were no significant differences in sex, age, diameter of the lesion, or position of the lesion between the AG- and PG-effective patients. Therefore, it remains difficult to identify AG-effective patients before a PET study.

We applied PG after AG in this study. FDG uptake is critically affected by the interval between FDG injection and data acquisition (13). This should be considered when evaluating the data obtained in the present study, as an additional scan for PG was conducted 15 min after the WB scan. To address this problem, we obtained the reference data (non-gated data) from the mid-point of the acquisition data in both methods. With the use of these internal references, two different scans with varying time intervals could be compared.

We used the best data of PG, whereas the conditions of AG were fixed. Grootjans et al. in their report suggested that a narrower bandwidth probably provides a better effect in the AG method (9). In our preliminary study, the AG data were generated from 20%, 25%, 30%, 35%, and 40% of the acquisition data, respectively.

Overall, data from smaller rates (e.g., 20% or 25%) showed better IR than higher rates (data not shown). This is one of the reasons we selected a 25% rate for AG. The second reason is that we aimed to compare AG and PG data over the same accumulation time (2 min), as similar conditions are desirable.

## Conclusion

We compared the SUV_max_ and IR values of PG and AG methods to clarify which method is preferable for measuring FDG accumulation in lung adenocarcinoma. We found that PG was more effective for measuring FDG accumulation of lung lesions under free-breathing conditions in comparison with the AG method. Therefore, PG should be selected primarily for most lung adenocarcinoma patients.
